# Droplet Lasers for Smart Photonic Labels

**DOI:** 10.1021/acsami.1c14972

**Published:** 2021-10-20

**Authors:** A. Capocefalo, E. Quintiero, C. Conti, N. Ghofraniha, I. Viola

**Affiliations:** † CNR ISC, Istituto dei Sistemi Complessi, c/o Università Sapienza, Piazzale Aldo Moro 5, 00185 Roma, Italy; ‡ CNR NANOTEC, Istituto di Nanotecnologia, c/o Università Sapienza, Piazzale Aldo Moro 5, 00185 Roma, Italy

**Keywords:** microlasers, whispering-gallery mode, photonic labels, anticounterfeiting, self-formation, viscoelastic dewetting, soft lithography, random lasers

## Abstract

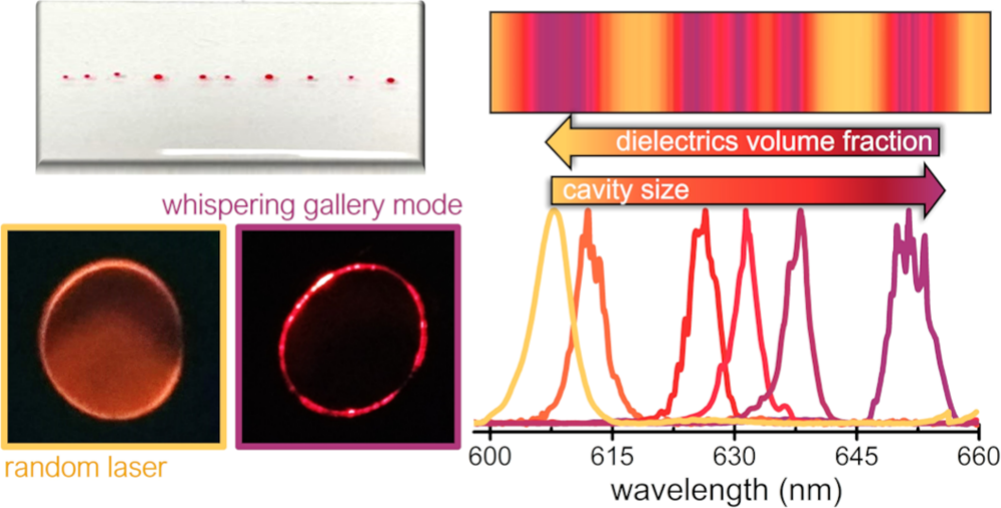

Microscopic lasers represent a promising tool for the development of cutting-edge photonic devices thanks to their ability to enhance light–matter interaction at the microscale. In this work, we realize liquid microlasers with tunable emission by exploiting the self-formation of three-dimensional liquid droplets into a polymeric matrix driven by viscoelastic dewetting. We design a flexible device to be used as a smart photonic label which is detachable and reusable on various types of substrates such as paper or fabric. The innovative lasing emission mechanism proposed here is based on whispering gallery mode emission coupled to random lasing, the latter prompted by the inclusion of dielectric compounds into the active gain medium. The wide possibility of modulating the emission wavelength of the microlasers by acting on different parameters, such as the cavity size, type and volume fraction of the dielectrics, and gain medium, offers a multitude of spectroscopic encoding schemes for the realization of photonic barcodes and labels to be employed in anticounterfeiting applications and multiplexed bioassays.

## Introduction

Microlasers that confine light in miniaturized cavities hold great potential in photonics and optoelectronics applications due to the capability to accurately probe small physicochemical variations of their microenvironment via light–matter interaction, providing a univocal optical readout.^1^ Moreover, their microscopic size allows them to be easily integrated in portable functional devices such as lab- or organ-on-chips. All these features meet the increasing demand of modern photonic technologies for better performances, sensitivity, low lasing thresholds, high spatial resolution, and cost-saving mass-manufacturing processes.^2,3^ The ease of configuration and signal detection make microlasers specifically suitable to be employed in applications in which miniaturized emitting devices with specific spectral features are required, as in multiplexed bioassays, anticounterfeiting labels, and material tracking.^4−6^

Further technologies with addressable optical response for the realization of stimuli-responsive sensors and antiforgery devices include the employment of dye-doped polymers with tunable photoemission^7−9^ and micro- and nano-structured materials with long-lasting fluorescence.^10−12^

Among the different options available, particularly captivating are microlasers relying on whispering gallery mode (WGM) emission, namely micron-sized optical cavities with a hollow geometry that, thanks to the phenomenon of total internal reflection, entrap light at their surface.^13,14^ The constructive interference of circulating light leads to the formation of sharp optical resonances whose wavelength depends both on the shape and size of the cavity as well as on the effective refractive index at the interface with the external medium.^15^

The embedding of a gain medium in the cavity together with the employment of a suitable pump source turns these microresonators into active cavities in which the spontaneous emission of the dye is modulated by the WGM resonances and eventually results in stimulated emission.^16^ In this way, the resonator behaves as a microscopic laser source with small mode volume and a low lasing threshold, that can reach high quality factors, Q, up to 10^11^.^15,17^

The high intensity of the WGM laser emission is extremely sensitive to infinitesimal changes at the resonator interface such as fluctuations of the effective refractive index or variations in the shape and size of the cavity. These changes result in measurable shifts of the resonance wavelength, and they can be thus tailored to design robust spectroscopic encoding schemes for the development of photonic barcodes^4,18^ as well as to detect molecular interactions in biological samples^19,20^ or changes of the environmental conditions.^21^ In addition, the wide choice of materials and morphologies available^13,22,23^ have made WGM lasers leading-edge devices for advanced applications in chemical sensing,^24−26^ optomechanics,^27^ and intracellular tagging.^3,28,29^

The most suitable geometry that allows us to reach considerable quality factors, and thus obtain high-performance lasing for applications, is a cavity with a smooth spherical surface that minimizes radiation losses. Liquid microspheres or microdroplets have been the subject of investigation since the very beginning of this field.^15^ Up to now, most WGM microcavities are made from solid materials with engineered geometries. Recently, novel and challenging fabrication techniques have relied on exploiting the fluidic nature of active materials to engineer photonic devices with tunable performances. This approach has allowed creation of scaffolds of hollow structures from the evaporation of liquid droplets^30^ or using the droplets themselves as cavities.^31−34^ For both approaches, the liquid interface between two immiscible fluid phases naturally forges smooth spherical surfaces thanks to an interplay of different surface tensions.^35,36^

Furthermore, liquid WGM microresonators open up to biosensing and imaging applications more easily than their solid counterpart. This is thanks to, for instance, the ability to intracavity assemble a variety of functional organic molecules or inorganic particles to modulate and improve light output.^19^ Nevertheless, although droplet-based WGM microlasers provide several advantages compared to other cavities, they can be challenging in terms of mechanical deformation, stability, and time duration.^22^

The fabrication of a three-dimensional (3D) integrated WGM microlaser made of a liquid droplet actually requires outstanding efforts for the integration with multiplexed devices, sample delivery, and data capture, as well as chemical stability of the functional material in the liquid cavity or addressable functionalization for specific molecule detection.^22^

In this work, we realize tunable and flexible-doping liquid droplet microresonators with WGM lasing emission that can be employed as multifunctional photonic labels with desired features. Such a liquid microlaser is obtained via the spontaneous self-formation of 3D active microdroplets of a gain solution in a liquid elastomer (polydimethylsiloxane, PDMS), at room temperature, by driving the surface and interfacial tensions of the different phases. The dye-doped microdroplets, located inside the elastomeric flexible matrix, have stable and shape-retaining spherical cavities suitable for WGM lasing emission.

The fabrication technique allows an in situ functionalization of the microdroplets and a real-time multimode analysis of the lasing behavior. The droplets of liquid are also characterized by an effective compartmentalization of the active materials through the self-assembly properties and by the possibility of varying sizes from hundreds of micrometers to a few millimeters.

We perform an optical sensing of the microresonators by gradually doping the liquid gain medium with organic and inorganic compounds with different refractive index and distributed in a random and unique way. The detailed analysis of the wavelength shift with respect to the variation of the refractive index of the cavity, induced by the doping particles, enables to identify the mechanisms contributing to light emission. The occurrence of two different lasing mechanisms, WGM and random laser (RL), allows us to obtain significative and distinct emission band shifts. Thanks to this tunable optical response, we realize smart lasing labels consisting of arrays of self-formed droplets in which each individual microlaser encodes a specific spectral fingerprint defined by the droplet’s size and volume fraction of the doping material. The average spectrum of the array gives one unique barcode for distinctive identification.

## Results and Discussion

### Fabrication of Liquid Droplet Microlasers

The fabrication of 3D WGM flexible microresonators is carried out by spontaneous, single-step, self-formation of a microsized droplet of hydrophilic doped liquid put in contact with a hydrophobic PDMS liquid matrix at room temperature. The difference in the energetic properties, at the surface interface among the two liquid phases, leads to the creation of microdroplets with tailored properties.

**Figure 1:**
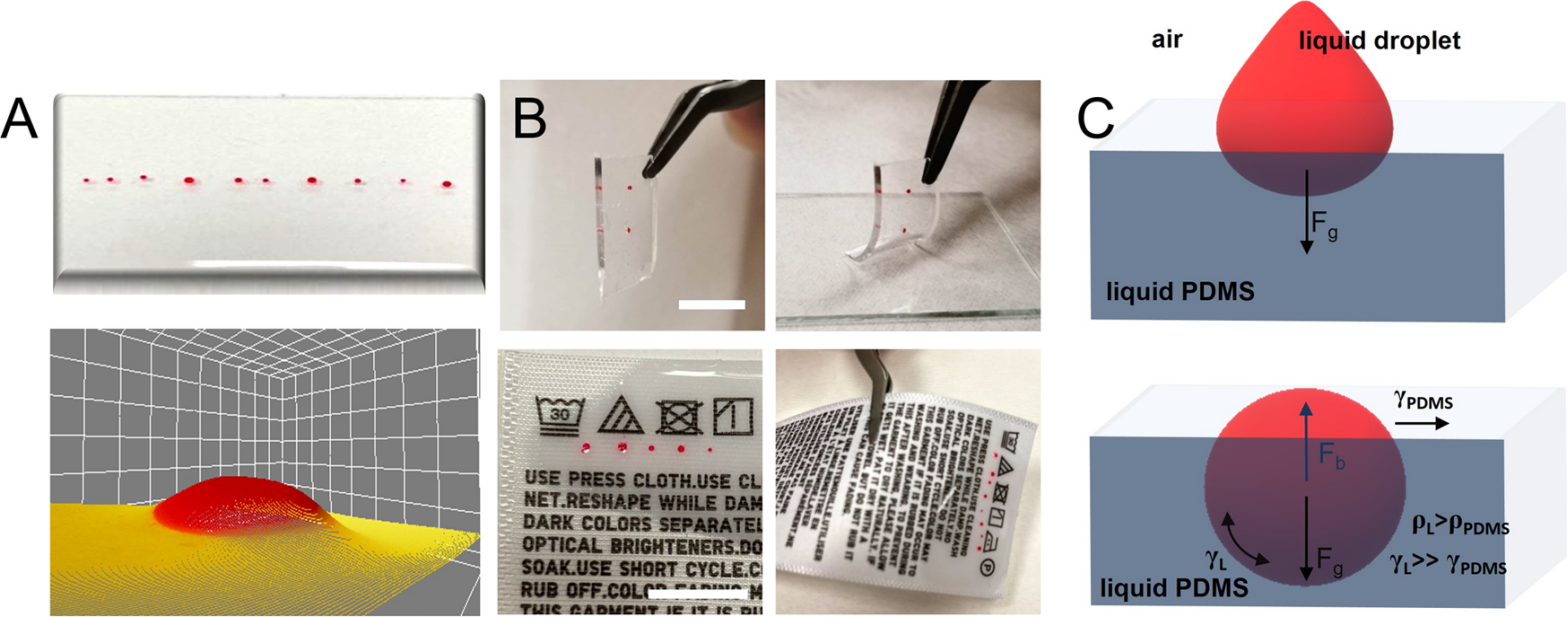
(A) Photograph of the smart WGM lasing device obtained via the spontaneous self-formation of 3D active microdroplets of a gain solution in a liquid elastomeric matrix. Such a polymeric device is realized by a random sequence of microdroplets characterized by different sizes and different compositions of dielectric particles included into the gain medium and compatible with a barcode reading. Below, the 3D reconstruction of the optical images of a droplet section outside the elastomeric matrix before encapsulation. (B) Images of the WGM device realized on a polymeric matrix (top) and on a clothes’ tag (bottom). The pictures show the free-standing structure of both labels which can be realized on multiple substrates, detachable and reusable. Scale bars correspond to 1 cm. (C) Spontaneous, single-step droplet formation due to a viscoelastic dewetting between two immiscible liquid phases: a gain solution of dye-doped diethylene glycol [rhodamine B (RhB) in DEG] and a liquid slab of polydimethylsiloxane (PDMS). The condition for a spherical shape of the lasing droplet is guaranteed by the density difference (ρ_L_ > ρ_PDMS_) and the interplay of surface tensions (γ_L_ ≫ γ_PDMS_).

As reported in Figure 1A,B, this approach allows the realization of functional devices within a polymeric matrix with a free-standing structure directly on the site of use (i.e., on paper sheet, fabrics, or glass), characterized by high flexibility and deformability. The device can be detachable and reusable on multiple substrates; it can be bent and rolled. A careful procedure avoids destroying or damaging the shape, symmetry, and entirety of each microdroplet. The fabrication is also possible on other types of substrates without changing the procedure (see Figure 1B).

The spontaneous formation of spherical microdroplets, with diameters in the range of 100–1000 μm, is driven by the balance of the energetic forces acting on the system liquid droplet–PDMS matrix (sketch in Figure 1C). This fabrication approach, compared to other methods, such as inkjet printing,^20,37^ has the main advantage of being simple and cost-effective, and it can be carried out with ordinary laboratory tools without the need for a specific setup.

In particular, the assembly of the liquid microdroplet reflects the balance of the free energy *G* for the formation of drops. We can describe the process as a sort of dewetting between two liquid phases in a viscoelastic regime, applicable to polymers.^38,39^ Therefore, in describing the spontaneous self-formation of microdroplets, different contributions in the total free energy of the system should be taken into account: the bulk free energy that accounts for enthalpic interactions, *G*_bulk_; the surface energies of the two liquid phases (droplet’s liquid and PDMS), *G*_surface_; the interface energy between them *G*_interface_; gravity which depends on liquid density, *G*_gravity_; and the buoyancy force that acts on the liquid droplet when it sinks into the PDMS matrix, *G*_buoyancy_:
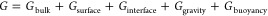
1

The development of the various terms of eq 1 suggests that the interplay between the density and surface tension of the drop solution and the liquid PDMS has a great influence on the encapsulation of the droplet within the flexible matrix.^30^ All the force terms involved in the formation of the droplet are shown in Figure 1C. Taking these considerations into account, we have carefully chosen the properties of the liquids to obtain the microlasers with the desired shape and composition. The theoretical model of Choi et al.^30^ suggests, in fact, that an interplay between the densities ρ of the different liquid phases and their surface tensions γ allows us to obtain liquid droplets with different properties. Here, we have appropriately selected the properties of different liquid phases in order to obtain spherical microdroplets, symmetrically embedded in the liquid PDMS. Specifically, we have used DEG (ρ_DEG_ = 1.12 g/mL and γ_DEG_ = 44.8 mN/m) and water (ρ_W_ = 1.00 g/mL and γ_W_ = 72.8 mN/m) for the liquid phases at room temperature. Uncured liquid PDMS (ρ_PDMS_ = 0.97 g/ml and γ_PDMS_ = 22–25 mN/m) was used as a flexible support matrix. Indeed, our findings attest that the required microdroplet occurs when the density of the liquid is greater than that of the PDMS, ρ_L_ > ρ_PDMS_, and its position within the flexible matrix is defined by the liquid density value. Similarly, the droplet shape and the condition of sphericity, mandatory for the WGM resonances, are guaranteed by a surface tension of the liquid much higher than that of PDMS, γ_L_ ≫ γ_PDMS_. In fact, considering the Young–Laplace equation^40^ in the *G*_surface_ term of eq 1, we can obtain an inequality on the contact angle θ value, −1 ≤ cos θ < 0. Therefore, the droplet takes an ellipsoidal shape for γ_L_ ≤ γ_PDMS_, while a spherical one for γ_L_ ≫ γ_PDMS_. The energetic conditions, thus defined, allow a reproducibility and stability in the droplet formation, which we have tested for diameters ranging from hundreds of microns to a few millimeters.

**Figure 2:**
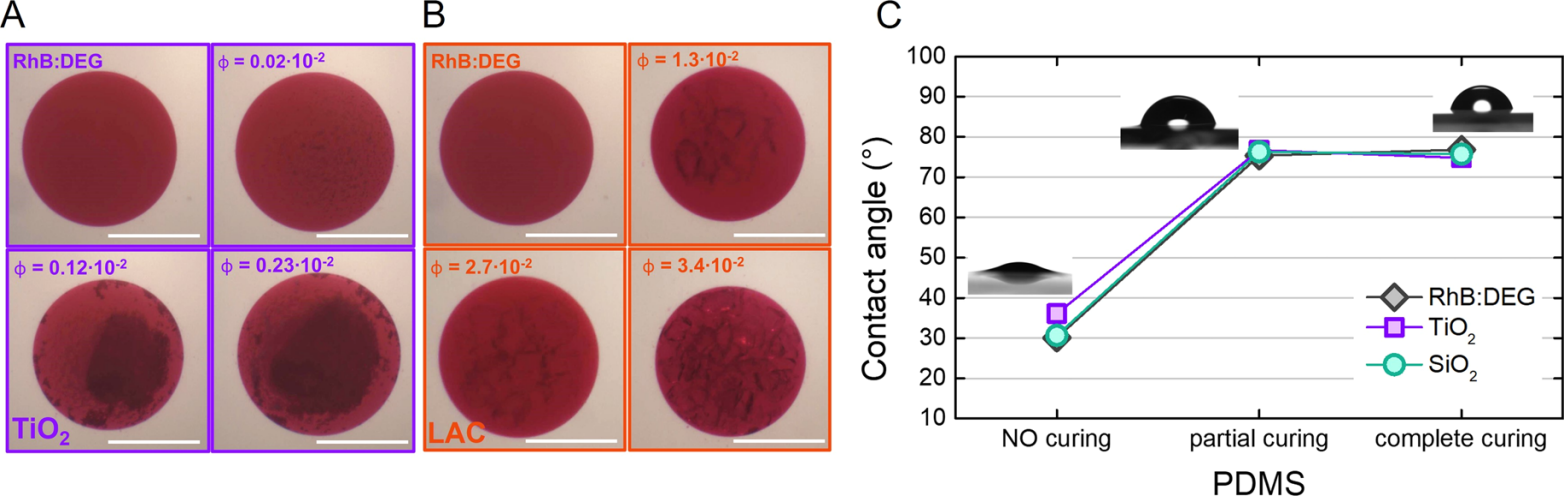
(A,B) WGM liquid microdroplets obtained by enriching the reference RhB:DEG solution with scattering materials at different concentrations. The pictures show functionalized microdroplets with increasing volume fraction ϕ of TiO_2_ (A) RhB:DEG, ϕ = 0.02 × 10^–2^, ϕ = 0.12 × 10^–2^, and ϕ = 0.23 × 10^–2^; and LAC (B) RhB:DEG, ϕ = 1.3 × 10^–2^, ϕ = 2.7 × 10^–2^, and ϕ = 3.4 × 10^–2^. The scale bars in the figures correspond to 500 μm. (C) Contact angle measurements of different DEG solutions (RhB:DEG; TiO_2_ + RhB:DEG; SiO_2_ + RhB:DEG) at the PDMS interface are reported. PDMS substrates at different stages of polymerization have been used to monitor the effect of the matrix on the droplet self-formation process.

We have used a solution of RhB in DEG (RhB:DEG@3 mM) as the reference liquid for the microcavity self-formation. Therefore, given the stability of the fabrication technique, and given the application as a tunable WGM microlaser, we have carried out self-formation of the microdroplets by enriching the reference RhB:DEG solution with different dielectric materials at different volume fractions. Table 1 summarizes the properties of the liquid used. In Figure 2, examples of the different microdroplets obtained are shown. Specifically, as dielectric compounds, we separately used (i) titanium-dioxide micropowder (TiO_2_), (ii) silica nanoparticles (SiO_2_), (iii) lactose monohydrate (LAC), and (iv) bovine serum albumin (BSA). Details are reported in the Experimental Section. All of these scatterers were added in different concentrations to the RhB:DEG solution in order to modulate the emission spectra of the final microlaser as a function of the volume fraction of the dielectric compounds (see Table 1). As an example, TiO_2_- and LAC-functionalized microdroplets at different volume fractions are shown in Figure 2A,B.

**Table 1:** Characterization of the Liquid Solutions Employed for the Formation of WGM Liquid Microdroplets in Terms of Solution Density and Volume Fraction of Dielectric Particles: TiO_2_, SiO_2_, LAC, and BSA

dispersion	density (g/cm^3^)	volume fraction ϕ (×10^–2^)
TiO_2_ + RhB:DEG	1.34	0.02
	2.20	0.12
	2.42	0.14
	3.27	0.23
SiO_2_ + RhB:DEG	1.17	0.50
	1.34	2.50
	1.56	5.00
LAC + RhB:DEG	1.12	0.20
	1.12	1.30
	1.13	2.70
	1.13	3.40
BSA + RhB:DEG	1.04	0.30
	1.07	1.40
	1.09	2.80

A good control on the diameter of the droplets, which defines the laser cavity size, was obtained by (i) exploiting imbibition phenomena through a microtip, driven by capillarity and surface tension of the liquid itself, for the smallest droplets with 100–200 μm diameter, and (ii) using a volume-controlled micropipette (Pipetman P2L, Gilson) for larger droplets with diameter greater than 200 μm.

The role of liquid properties in the droplet formation has been preventively evaluated by monitoring the contact angle of different DEG solutions at the PDMS interface. PDMS substrates at different stages of polymerization, from freshly prepared PDMS at room temperature (liquid stage) to completely polymerized PDMS (solid stage), have been used for this purpose. The results in Figure 2C (and Table S1 in the Supporting Information) show that the component of scattering materials does not affect the energetic behavior at the interface. On the contrary, the degree of PDMS polymerization plays a crucial role in the dynamics and quality of droplet self-formation. Even a partial or a complete curing of the PDMS does not allow the drop to be incorporated into the polymeric matrix while maintaining a spherical drop shape. The liquid PDMS instead allows the drop to sink within the polymer matrix while maintaining high contact angles (θ > 70°), which give sphericity to the droplet. Finally, to prevent evaporation of the solvent and keep the microresonators stable, the self-formed microdroplets were encapsulated with a thin layer of PDMS. Such a multilayer device proved to be functional and stable for several weeks.

### Laser Emission of RhB in DEG Microdroplets

**Figure 3:**
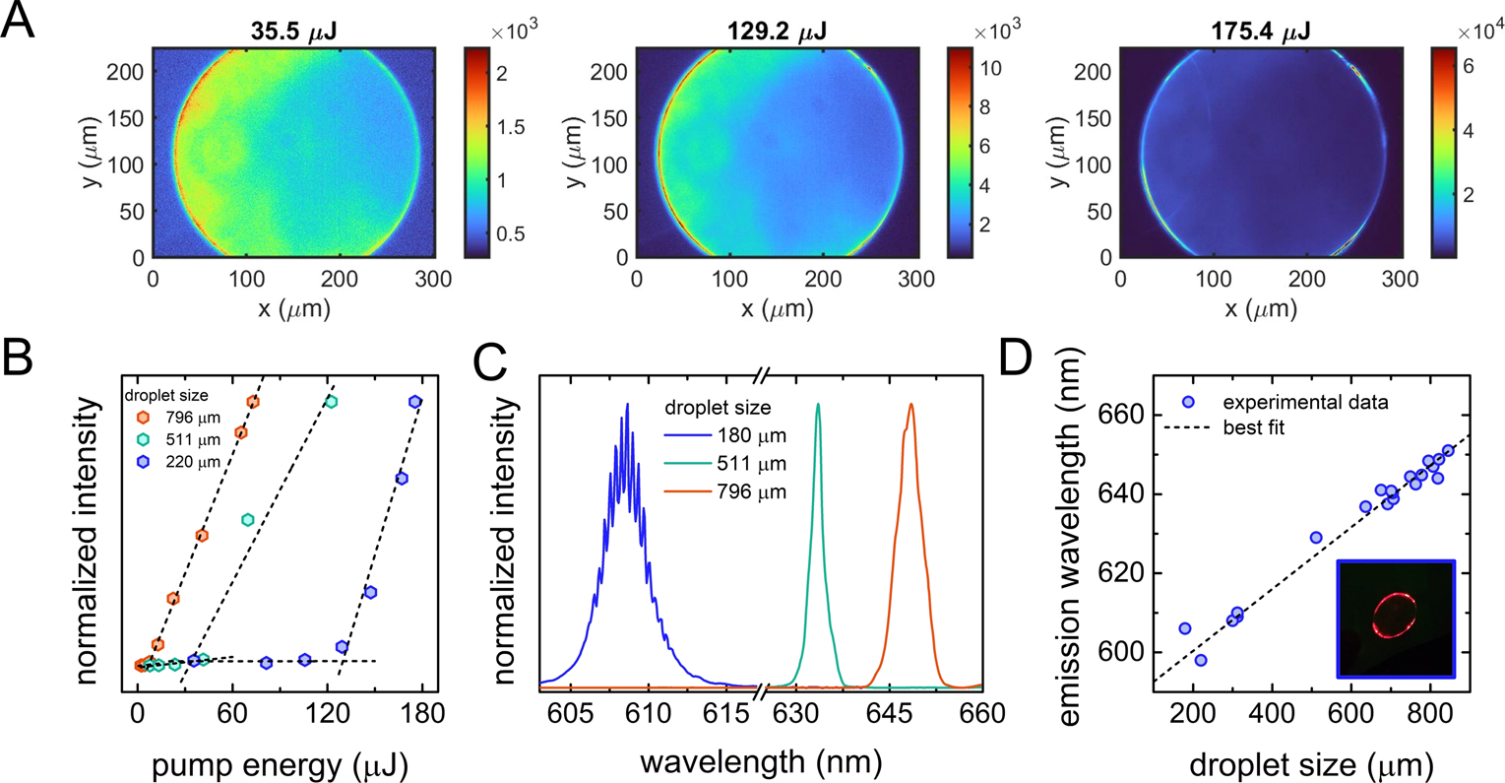
(A) Fluorescence images of a RhB:DEG droplet at varying pump energy. The first two images are below the lasing threshold, while the third image is above the lasing threshold. (B) Representative trends of peak intensity as a function of pump energy for three RhB:DEG droplet lasers with different diameters. Dashed lines are the linear fitting curves. The estimated energy thresholds are in the range 8–130 μJ, depending on the droplet diameter. (C) Representative emission spectra of RhB:DEG droplets with different sizes. For the droplet with smaller size, the periodic peaks typical of WGM lasing emission are evident in the spectrum. At increasing droplet size, the spectrometer resolution does not allow us to resolve the single lasing peaks. (D) Central wavelength of the emission spectra as a function of the RhB:DEG droplet size. The dotted line is the linear fitting curve. The inset shows a magnified picture of a RhB:DEG droplet in which the intense fluorescence at the droplet’s rim is evident.

The laser emission characterization of RhB in DEG liquid droplets, obtained by employing the optical setup described in the Experimental section, is reported in Figure 3.

The emission phenomenology of a single liquid droplet is evident from the fluorescence images at varying input energy reported in panel A. Below the lasing threshold (35.5 μJ in Figure 3A), we observe spontaneous emission uniformly distributed over the entire droplet whose efficiency is determined by the fluorescent dye. At increasing input energy, the emission intensity increases significantly. Above the onset of lasing (175.4 μJ in Figure 3A), the emitted light is localized at the rim of the sphere, with higher intensity with respect to the center of the droplet. This behavior is indicative of the activation of radial resonant modes typical of WGM resonant cavities with axial symmetry.^13,22^

Three representative trends of the emission intensity as a function of the pump energy obtained for droplets with different sizes are depicted in Figure 3B, showing the two regimes of spontaneous and stimulated emission. The energy thresholds corresponding to the onset of lasing are determined from the intersection of the two linear regimes and fall in the range 8–130 μJ for all the droplets analyzed with variations that depend on the droplet size. Specifically, a droplet with smaller size contains a smaller volume of excitable gain medium and thus, the resonance efficiency of the radial cavity is lowered.^41^

Representative emission spectra corresponding to RhB droplets with different sizes are reported in Figure 3C. The emission is shifted toward smaller wavelength with decreasing the cavity size, spanning in a broad spectral range determined by the RhB fluorescence emission band.

An accurate analysis of the emission wavelength at varying droplet diameters in the range 150–900 μm is reported in Figure 3D. The central wavelengths of the emission spectra follow a linear growth with the droplet size, according to WGM theory^13,14,17,42^
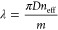
2where λ is the central emission wavelength, *D* is the cavity diameter, *n*_eff_ is the effective refractive index of the cavity, and *m* is an integer number that corresponds to the azimuthal quantum number of the mode. Noteworthily, the free spectral range (FSR) as well shows a dependence on the cavity size *D*, given by the relation^13,43^ FSR = λ^2^/π*Dn*_eff_. In Figure 3 C, the periodic oscillations typical of WGM lasers are evident for small-size droplets. For droplets with a diameter from 500 μm and above, as those employed in this work, it is not possible to resolve the single WGM lasing peaks due to the spectral resolution of the spectrometer. In fact, in this case, the FSR is comparable to the sampling rate of the instrument (given a spectral resolution of 0.07 nm, the minimum detectable full width at half-maximum (FWHM) of a peak is ∼0.14 nm); therefore, the periodicity of the WGM lasing peaks is distorted.

The elastomeric nature of PDMS allows us to mechanically deform the device containing the liquid microlaser. We verify the lasing performances of a device with RhB:DEG droplets undergoing mechanical stress by stretching and bending it, as reported in section S2 of the Supporting Information. The lasing action of the device is retained under mechanical stress, and we observe in both cases a shift of the emission wavelength due to the change of the geometry of the optical cavity from a sphere to an ellipsoid. In view of employing the device as a photonic tag, the flexibility of the PDMS allows us to adjust it even on curved surfaces without preventing the lasing performances.

### Emission Mechanism of Microdroplet Lasers Doped with Dielectric Materials

Here, we explore the possibility of tuning the WGM emission by finely changing the internal refractive index of the cavity by doping the liquid RhB:DEG droplets with dielectric compounds showing different refractive indices. Specifically, we employ TiO_2_, SiO_2_, LAC, and BSA (see Table 1). In order to have a broad spectral range for the modulation of the emission properties of the microresonators over the whole emission band of RhB, in the following study, we selected liquid droplets with diameters larger than 500 μm.

**Figure 4:**
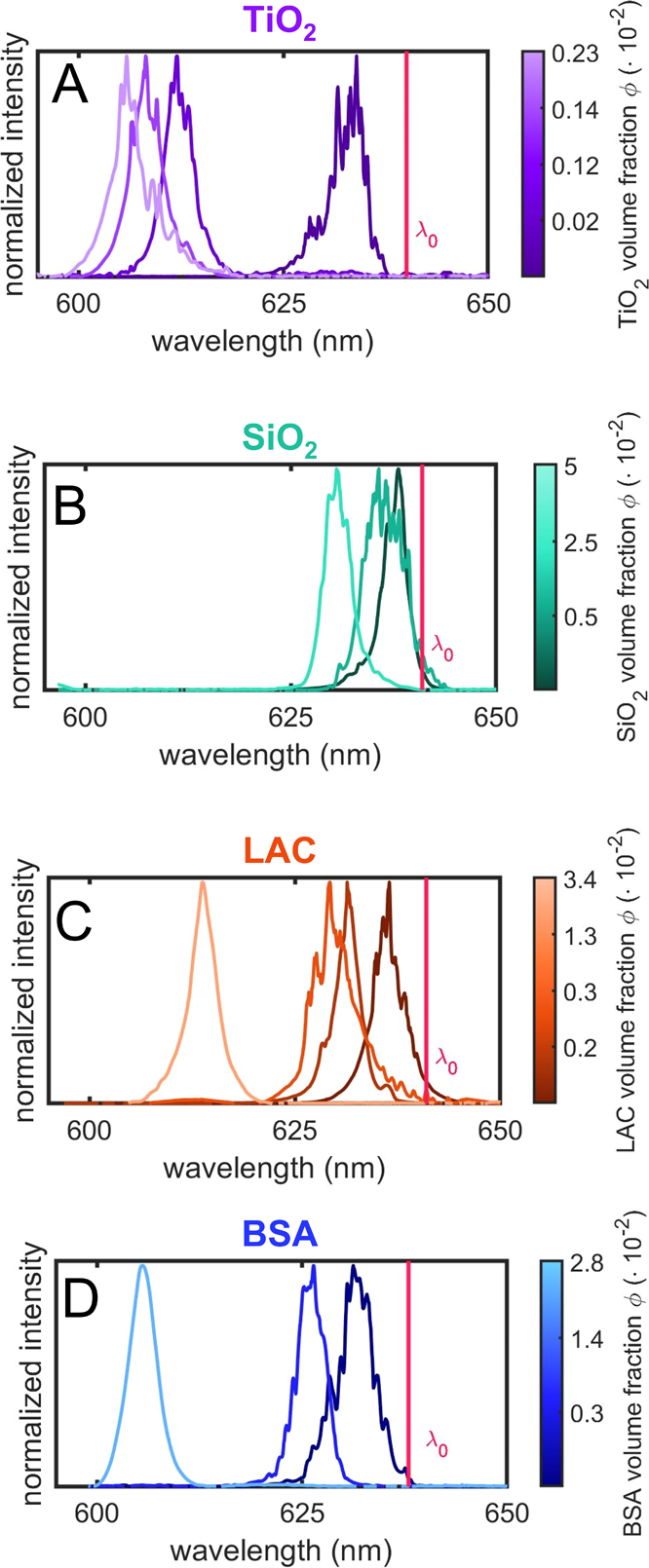
Emission spectra of droplet microlasers at varying volume fractions ϕ of the doping particles: (A) TiO_2_, (B) SiO_2_, (C) LAC, and (D) BSA. The vertical line represents the emission wavelength λ_0_ of RhB:DEG droplets reported as the reference. All the spectra are normalized to the maximum intensity value.

The emission spectra of the microlasers at varying volume fractions ϕ of doping dielectric particles are reported in Figure 4A–D. All the samples show a progressive blue-shift of the emission wavelength with increasing concentration of the dielectric compounds. We observe that the extent of the wavelength shift and the spectral features depends both on the material and on the volume fraction of the particles. Specifically, TiO_2_-doped droplets (Figure 4A) show a marked blue-shift in the emission wavelength even at a small volume fraction of particles, while in the case of SiO_2_-doped droplets (Figure 4B), the observed wavelength shift is lower despite the higher volume fraction of SiO_2_ particles. The emissions of LAC- and BSA-doped droplets (Figure 4C,D) follow a similar behavior as a function of the volume fraction, with emission spectra that become narrow and smooth at the maximum volume fraction of particles.

In order to better quantify the lasing behavior, we calculate the spectral shift Δλ between the emission wavelength λ_0_ of the RhB:DEG droplets with respect to the λ_D_ of the doped droplets. The emission wavelength λ_D_ is the central wavelength of the emission spectra. The wavelength λ_0_ of the reference for each droplet was determined from the linear fit reported in Figure 3D in order to take into account the wavelength shift owing to the droplet diameter *D* (see equation 2), according to the formula λ_0_ = *q* + *s*·*D*, where *q* and *s* are the intercept and slope extrapolated by the curve fitting, respectively. Moreover, for the samples containing SiO_2_ and BSA particles that are dispersed in water, the slope was properly corrected considering the change of the refractive index of DEG due to the presence of a volume percentage of water:  where *n*_DEG+H_2_O_ is calculated from the volume percentage ϕ_w_ of water according to the equation
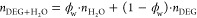
3where *n*_H_2_O_ = 1.33 and *n*_DEG_ = 1.447.^44^

We collected spectra for all the typologies of dielectric-doped droplets and the Δλ values reported in the following correspond to the mean and standard deviation of the central emission wavelength measured for the different samples at a given volume fraction of particles.

**Figure 5:**
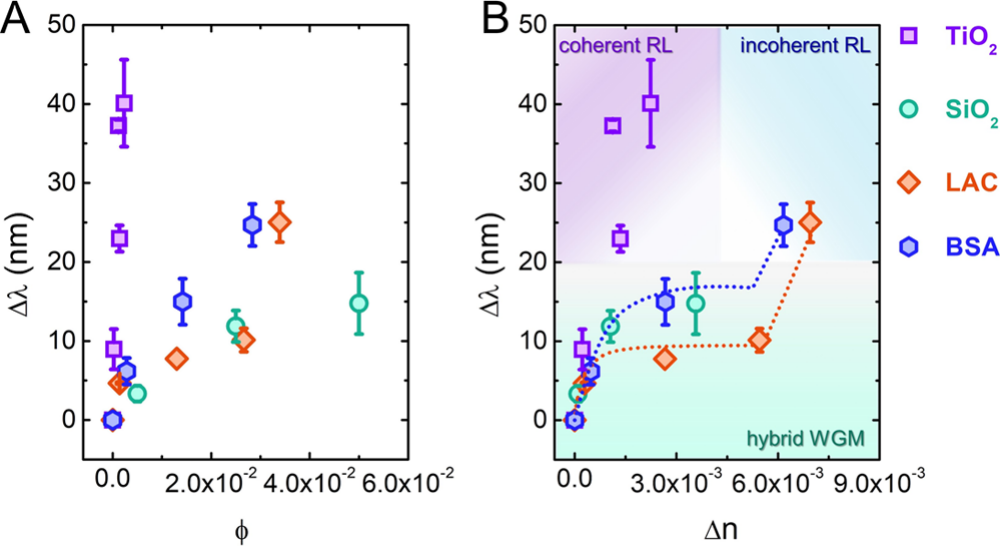
Dependence of the emission wavelength on the amount of dielectric particles: TiO_2_, SiO_2_, LAC, and BSA. (A) Wavelength shift as a function of the volume fraction ϕ of doping particles. (B) Wavelength shift as a function of the variation in the effective refractive index of the doped droplets with respect to that of droplets obtained by pure RhB:DEG. In the figure, the three different mechanisms hypothesized for the emission are represented. The dotted lines in the figure are a guide for the eye.

The spectral shift Δλ as a function of the volume fraction ϕ of doping particles is reported in Figure 5A. The data highlight a progressive shift of the emission as a function of the volume fraction, with the maximum wavelength shift that depends on the material. In particular, we observe a maximum shift of 40 nm in the case of TiO_2_-doped droplets and a maximum shift of 14 nm for SiO_2_-doped droplets despite the volume fraction that is 1 order of magnitude higher.

Given the crucial role of the variation of the internal refractive index of the cavity in the modulation of the emission property of the microresonators, we further evaluate the spectral shift Δλ with respect to the variation of the effective refractive index induced by the doping of the RhB:DEG droplets. The variation Δ*n* of the effective refractive index of the RhB:DEG droplets with respect to the doped droplets is calculated according to the equation
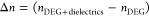
4where *n*_DEG+dielectrics_ = ϕ·*n*_d_ + (1 – ϕ)·*n*_DEG_, where ϕ and *n*_d_ are respectively the volume fraction and the refractive index of the dielectric compound. In the case of SiO_2_ and BSA-doped droplets, *n*_DEG_ is replaced with *n*_DEG+H_2_O_ calculated from eq 3.

**Figure 6:**
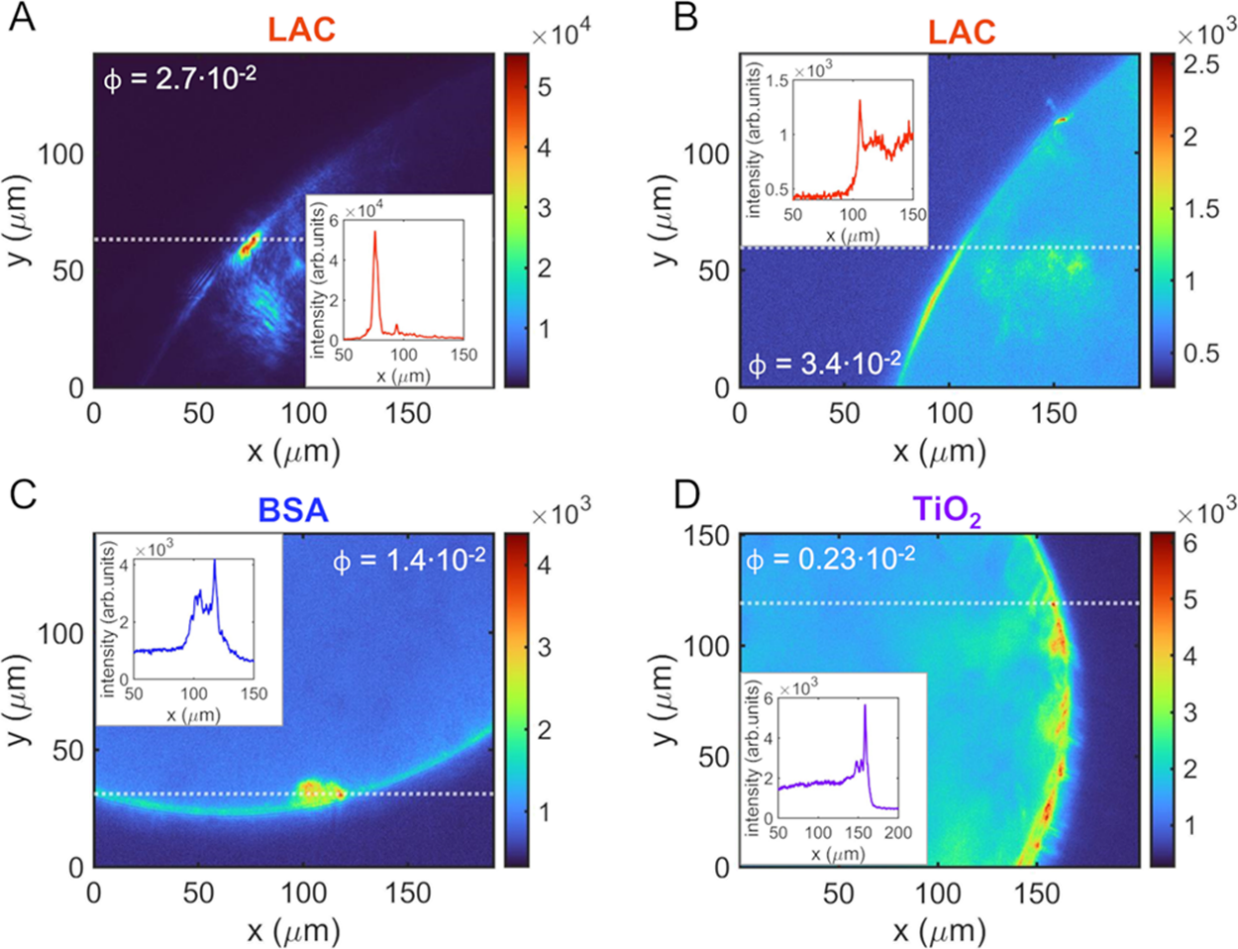
Fluorescence images of different doped droplets. LAC + RhB:DEG droplets at two different volume fractions: ϕ = 2.7 × 10^–2^ (A) and ϕ = 3.4 × 10^–2^ (B). BSA + RhB:DEG droplet at ϕ = 1.4 × 10^–2^ volume fraction (C). droplet at 0.23 × 10^–2^ volume fraction (D). The insets in the figures represent the profile of the fluorescence intensity corresponding to the section indicated by the dotted lines.

The spectral shift Δλ as a function of Δ*n* is reported in Figure 5B. The nonlinear trend of Δλ suggests that beyond WGM lasing, an additional optical mechanism can contribute to the emission of the microlasers. In this respect, the direct visualization of the emitted fluorescence allows us to better understand the trends observed in the emission spectra. Representative fluorescence images of different doped droplets are reported in Figure 6. In the case of LAC-doped droplets, for the lowest concentration (Figure 6A), the emission is dominated by the presence of hot spots with high intensity in correspondence with aggregates of particles inside the droplet. At the maximum volume fraction (Figure 6B), we observe a marked decrease of the overall fluorescence intensity pointing out a breaking of the cavity symmetry. We observe the same behavior for BSA- and TiO_2_-doped droplets, reported in Figure 6C,D, respectively. SiO_2_-doped droplets instead do not show significant differences in the spatial distribution of the emitted fluorescence with respect to RhB:DEG droplets.

The observed behavior points out that the dielectric particles included in the droplets, besides gradually modifying the droplet’s internal refractive index, may assemble into aggregates that act as sites suitable for the light confinement.^45,46^ More specifically, such a system made up of a gain medium enclosing disordered ensembles of particles is defined as RL.^47^ The aggregates behave as centers for the multiple scattering of light that above a certain pump threshold results in stimulated emission. Depending on the nature and structure of the scattering elements, RL emission can have both coherent (resonant) and incoherent (nonresonant) lasing feedback.^48^ The first one is characterized by emission spectra with multiple lasing peaks, while the second one results in a single wide spectral peak.^49−53^

The optical behavior of the doped droplets suggests a progressive transition of the stimulated emission from the WGM toward a RL emission with increasing particle concentration, by passing through a hybrid emission mode where the two mechanisms coexist. At low volume fractions, the particles assemble into aggregates whose size is small with respect to the droplet diameter, and therefore, the fluorescent light is reflected unhindered multiple times across the rim of the droplet. This results in an efficient WGM emission that is added to the RL emission originating from the multiple scattering of light within the aggregates. Conversely, at the highest volume fractions, the size of the aggregates increases, and these bigger aggregates obstruct the optical path hindering the complete reflection of light around the rim of the droplet and thus resulting in the suppression of the WGM emission. In this case, we observe the typical spectral signature of incoherent RL emission, as reported for LAC- and BSA-doped droplets. In the case of TiO_2_ droplets instead, the high refractive index of TiO_2_ implies that even at very low concentrations, the main mechanism is a coherent RL emission. Noteworthily, the morphology of the aggregates, in terms of the packing fraction and roughness, plays a crucial role in determining the spectral features of the random lasing emission.^50,53^

The presence of dielectric compounds affects the lasing performances also in terms of the energy threshold as reported in section S3 of the Supporting Information. We observe a slight increase in the energy thresholds in the case of LAC, SiO_2_, and BSA, while for TiO_2_-doped droplets, the lasing threshold increases remarkably due to the higher losses of the disordered cavity. In addition, line narrowing, quantified by the abrupt decrease of the spectral FWHM (reported in the Figure S3), is more pronounced in the presence of TiO_2_.

With reference to Figure 5B, for spectral shifts within 20 nm, we ascribe the emission to a hybrid mechanism to which both the WGM and RL contribute, characterized by the presence of RL hot spots. Rather, for a larger wavelength shift, the main emission mechanism is owing to random lasing. In particular, TiO_2_-doped droplets are characterized by a coherent RL emission; indeed, the spectra display the features of multimode emission typical of RL with coherent feedback. Conversely, for LAC- and BSA-doped droplets, we observe the onset of incoherent RL emission, which is highlighted by the steep variation of Δλ versus Δ*n*.

On the basis of the data reported in Figure 5A,B, it is possible to obtain an estimate of the limit of detection (LOD) of the droplet resonators both for the analyte concentration and for the consequent variation of the refractive index, as reported in section S4 of the Supporting Information. The values of minimum detectable concentration range from 16 μg/mL to 1 mg/mL depending on the material. The measured LOD values for variations of the refractive index, expressed in refractive index units (RIU), range from 6.3 × 10^–6^ to 1.3 × 10^–5^ RIU. These values correspond to sensitivities in the range 6250–33,300 nm/RIU. The obtained sensitivity values are higher with respect to those measured for similar systems, such as 530 nm/RIU for water-based microdroplets,^54^ 500 nm/RIU for SiO_2_ droplets,^55^ and 160 nm/RIU for quantum-dot-embedded polystyrene microspheres.^56^ The high sensitivity to small amounts of dielectric particles included into the active medium opens the way to the employment of such microlasers as ultrasensitive sensor devices for the detection of various types of low-concentration analytes in solution. On top of this, the possibility to handle the microcavity lasing emission mechanisms, in addition to the flexibility provided by the fabrication technique, allows us to realize miniaturized laser emitting devices with a wide choice of optical encoding schemes to be used as smart lasing labels.

**Figure 7:**
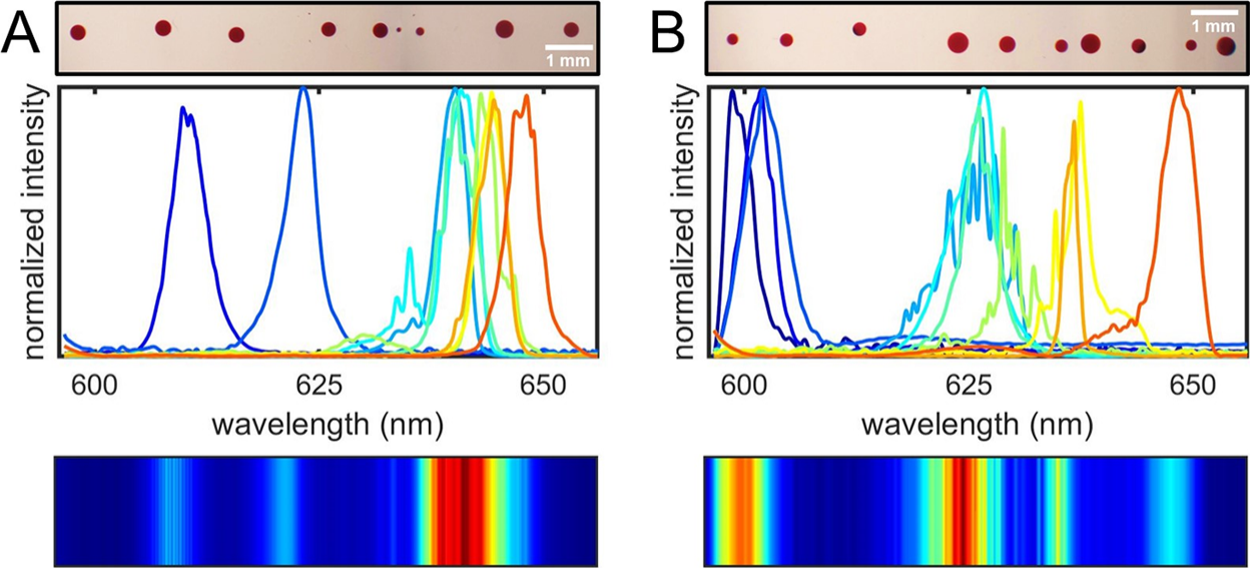
Examples of two barcodes realized with liquid microdroplet lasers. For each sample, the image acquired with a 4× magnification, the emission spectra acquired sequentially, and the corresponding barcode are reported. (A) Barcode realized with RhB:DEG microlasers at varying droplet size. (B) Barcode realized with RhB:DEG, TiO_2_ + RhB:DEG and LAC + RhB:DEG microlasers at varying droplet size and volume fractions of the dielectric compounds.

As a proof-of-concept to demonstrate the actual applicability of the proposed device for the realization of photonic labels, we realize two different barcodes that consist of a sequence of microdroplet lasers with different features, as reported in Figure 7. The first barcode is fabricated by employing the RhB:DEG solution as gain medium and the size of the microlasers is randomly varied along the sequence. The second one is realized by employing TiO_2_ + RhB:DEG and LAC + RhB:DEG as active media in addition to RhB:DEG, and we vary both the size of the microlasers and the volume fraction of TiO_2_ and LAC. The barcodes are read by exciting in sequence the microlasers and the succession of measured spectra defines the identification code. A numerical value corresponding to the intensity of the emission spectrum is associated to each wavelength, and the pattern of the barcode is obtained from the map of the average intensity calculated over all the spectra.

The decryption of the code can be easily achieved by comparing the pattern of the selected barcode with those corresponding to a specific product in the database. A simple parameter that allows us to quantitatively estimate the similarity between two patterns *i* and *j* is the Pearson correlation coefficient *C*_*ij*_,^21,53^ that is essentially a normalized measurement of the covariance between data sets *i* and *j*, and it is equal to 1 for identical patterns and equal to 0 for completely diverse ones. Given the uniqueness of each single barcode, the barcode of a certain product to be decoded will have high correlation (i.e., 0.9 ≤ *C*_*ij*_ ≤ 1) with the one registered in the database. The two barcodes of Figure 7 have *C*_*ij*_ = 0.16, a value close to zero proving that each code is unique and defined by the sequence of the single microlasers.

The wide possibility of varying both the size of the microdroplets and the type and volume fraction of the dielectric compounds dispersed in the gain medium offers infinite encoding schemes that are hard to counterfeit. The joint laser processes of the WGM and RL allow the fabrication of an array of microlasers with emission lines well separated in a wide spectral range of ∼40 nm and a unique landscape. Moreover, it is even possible to extend the spectral region of interest by employing an active dye different from RhB.

## Conclusions

In this work, we have exploited the self-formation of liquid droplets, driven by viscoelastic dewetting, to obtain smart photonic labels consisting of microlasers embedded into a polymeric matrix.

The fabrication of such functional WGM droplet microlasers is a spontaneous process due to dewetting phenomena between two immiscible viscoelastic liquid phases and is carried out at room temperature without any external force. We thus succeeded in the realization of smart and functional optical devices directly on the site of use, characterized by high flexibility and deformability. As a result, the devices obtained are ready to use, self-consistent, resistant, moldable, and easily adaptable according to the final interface or substrate. Given the adhesion and conformability properties of the PDMS, the self-formed droplet-based devices are extremely flexible and can be moved and reattached as a functional label, retaining the lasing activity.

The self-formation of liquid droplets within an elastic matrix allows high-precision lithography of directly functionalized solutions. The dimension and resolution of each resonator can be finely improved by using a precision microspotter.

We carried out a detailed characterization of the lasing emission properties of the droplets in terms of the emission wavelength and energy threshold as a function of the cavity size. We finely tune the spectral emission of such microresonators by incorporating compounds with different refractive index into the liquid gain medium at varying volume fractions. This process results in the progressive blue shift of the central emission wavelength of the microresonators and in the variation of the spectral features of emission spectra at increasing concentration of doping particles. The direct visualization of the emitted fluorescence from the droplets enabled to clarify the role of the particles dispersed inside that besides gradually modifying the effective refractive index of the cavity, they create efficient sites for the light entrapment. This determines the formation of disordered microscopic cavities embedded in the droplets that contribute and enhance the overall emitted intensity through random lasing hot spots. The switching from a hybrid emission mechanism to a pure RL emission depends on the nature of the particles and on their volume fraction that through the formation of aggregates determine a breaking of the spherical symmetry of the whispering gallery cavity.

The response dependence of the WGM laser emission on the size and composition of the single microdroplet, together with the flexibility in the lithographic technique, opens wide perspectives in the use of such devices for optical encryption and biosensing applications as smart labels and patches with barcode and QR code-based readings, also suitable to be integrated in multiplexed lab-on-chips as innovative light sources. Our approach in the design of the device provides indeed multiple robust encoding schemes for the realization of photonic barcodes due to the availability of different dyes to be employed as gain medium together with the opportunity of modulating the type and amount of doping material as well as the cavity size.

## Experimental Section

### Liquid Microdroplet Fabrication

To prepare the dye-doped reference solution, we dissolved RhB (C_28_H_31_ClN_2_O_3_) powder in DEG (C_4_H_10_O_3_) in order to obtain a 3 mM RhB:DEG solution. This solution was then used as such or added to other dielectric materials. Thereafter, we realized an uncured PDMS ((C_2_H_6_OSi)_*n*_) elastomer matrix (Dow Corning, Sylgard 184, 10:1) on a glass coverslip. We waited a few minutes in order to let it stabilize on the glass and, more importantly, to obtain a viscous and sticky consistence. This phase is crucial in order to have the right viscoelasticity for the PDMS matrix, to let the droplets accommodate and be properly incorporated within liquid PDMS. Afterward, a small volume of the desired RhB-based solution is deposited on top of the PDMS slab. After a complete stabilization, each slab was sealed by pouring some more uncured PDMS. At this point, the slabs with the encapsulated droplets were left to fully polymerize at room temperature for almost 24–48 h.

As dielectric compounds to be added to the RhB:DEG solution, we used (i) titanium-dioxide micropowder (TiO_2_, Sigma-Aldrich) in DEG (20 mg:1 mL), (ii) silica nanoparticles (SiO_2_, monodisperse in water with 170 nm particle size, PolyScience), (iii) lactose (monohydrate, LAC) dissolved in DEG (8 mg:1 mL), and (iv) bovine serum albumin (BSA, Sigma Aldrich) in aqueous solution (8 mg:1 mL). These solutions were added to the RhB:DEG one in different volumes in order to evaluate the modification in the emission spectra by varying the volume fraction of each scattering particles inside the RhB:DEG solution (see Table 1 and Figure 2).

### Contact Angle Measurements

To better understand dewetting phenomena involved in the fabrication process, a complete characterization of the interfacial energy of different liquid phases has been carried out by measuring contact angles (OCA20, DataPhysics) of the different solutions on PDMS slabs. We characterized the most concentrated solutions for every kind of scattering particle employed (ϕ_TiO_2__ = 0.23 × 10^–2^, ϕ_SiO_2__ = 5 × 10^–2^, ϕ_LAC_ = 3.4 × 10^–2^, and ϕ_BSA_ = 2.8 × 10^–2^) as well as PDMS slabs in three different stages of polymerization. In particular, we used uncured PDMS (liquid PDMS), a partially cured PDMS, and a completely polymerized one (solid PDMS). We observed a clear effect of the PDMS viscoelasticity on the RhB solution’s contact angle. See Figure 2C and Table S1 in the Supporting Information for complete results.

### Optical Setup

The liquid droplets were excited by a *Q*-switched Nd:YAG pulsed laser operating at 532 nm, with 4 ns pulse duration, and 10 Hz repetition rate. The input energy of the laser was modulated by a variable neutral density filter mounted onto a motorized stage. The pump light was then split in half through a beam splitter, and the reflected part was measured by an energy meter to monitor the pump energy variations. The transmitted beam was focalized with an objective on the sample with a spot size of ∼5 mm in order to excite the whole droplets. The emission from the single droplets was magnified with 40× magnification by an objective with NA = 0.4 and filtered by a notch filter to remove residual pump light. The filtered light was then split on a charge-coupled device (CCD) camera for fluorescence imaging and focused onto an optical fiber connected to a spectrometer with a spectral resolution of 0.068 nm equipped with a CCD array detector. All the light emitted by the whole droplet was collected. A sketch of the setup is reported in ref (53).

## Supporting Information Available

Contact angle measurements, study of the lasing emission upon mechanical stress, energy thresholds of microdroplet lasers doped with dielectric materials, and evaluation of the limit of detection (PDF)
