# Supplementary material for: Droplet Lasers for Smart Photonic Labels
Source: ACS Appl Mater Interfaces. 2021 Oct 20;13(43):51485–94. doi: 10.1021/acsami.1c14972 (PMC9296018; doi:10.1021/acsami.1c14972)
Supplement: Supplementary file 1 — am1c14972_si_001.pdf [file am1c14972_si_001.pdf]

Supporting Information

Droplet lasers for smart photonic labels

A. Capocefalo, [*,†] *E. Quintiero,* [‡] *C. Conti,* [†] *N. Ghofraniha,* [†,¶] *and I. Viola*[‡,¶]

[†]CNR ISC, Istituto dei Sistemi Complessi, c/o Università Sapienza, Piazzale Aldo Moro 5, 00185 Roma, Italy

[‡]CNR NANOTEC, Istituto di Nanotecnologia, c/o Università Sapienza, Piazzale Aldo Moro 5, 00185 Roma, Italy

[¶]These authors contributed equally to this work.

*E-mail: angela.capocefalo@uniroma1.it

S1. Contact angle measurements

Table S1. Values of contact angles of different DEG solutions (RhB:DEG; TiO_2+RhB:DEG; SiO_2+RhB:DEG) at PDMS interface are reported. PDMS substrates at different stages of polymerization (No curing; partial curing and complete curing) have been used to monitor the effect of matrix on the droplet self-formation. The instrumental error on each value is about 1% .

	**RhB:DEG**	**TiO_2+RhB:DEG**	**SiO_2+RhB:DEG**
**No curing**	30.10	36.10	30.70
**Partial curing**	75.35	76.70	76.30
**Complete curing**	76.83	74.80	75.70

S2. Study of the lasing emission upon mechanical stress

We here verify the lasing performances of a device containing RhB:DEG droplets undergoing both stretching and bending, as reported in Figures S1 and S2, respectively.

Given the flexibility of the polymer matrix made of PDMS and the liquid nature of the microdroplet laser, when the device is subjected to a mechanical stress the shape of the liquid droplet changes accordingly to the direction of the deformation. Despite this, the lasing action of the device is retained in both the cases analyzed. We observe a blueshift of the lasing emission wavelength due to the modification of the geometry of the optical cavity from sphere to ellipsoid.

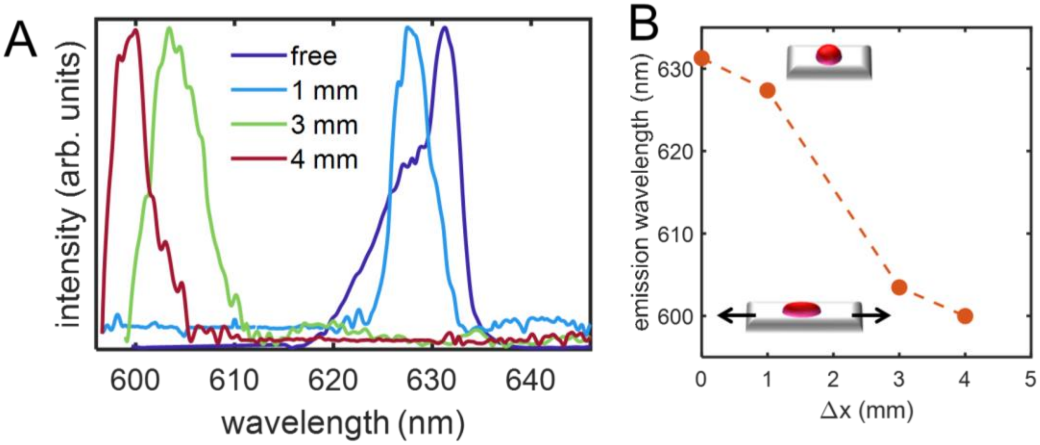

Figure S1. Study of the lasing emission of a RhB:DEG microdroplet laser undergoing longitudinal stretching. **A** Emission spectra at varying the stretching length. **B** Central emission wavelength as a function of the stretching length Δx.

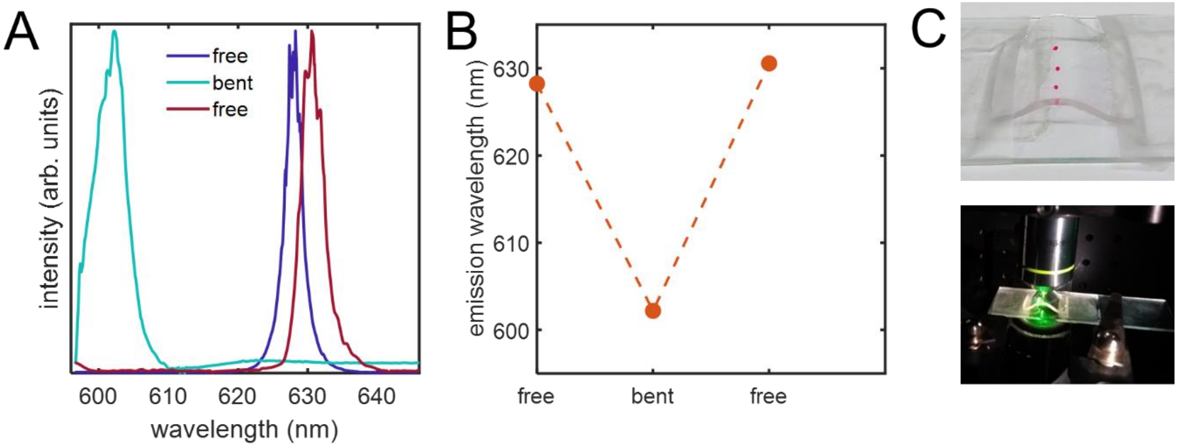

Figure S2. Study of the lasing emission of a RhB:DEG microdroplet laser undergoing bending. **A** Emission spectra of the free device (blue line), of the bent device (green line) and of the device released after bending (red line). **B** Central emission wavelength corresponding to the three configurations reported in Figure A. **C** Images of the bent device (top) and of the bent device illuminated by the laser source (bottom).

S3. Energy thresholds of microdroplet lasers doped with dielectric materials

The emission spectra of a RhB:DEG microlaser compared to those of a TiO_2+RhB:DEG microlaser at varying the pump energy are reported in Figure S3. To resolve the full emission band of the RhB dye the spectra were acquired with a 600 gr/mm diffraction grating. The trend of the emission peak intensity and that of the spectral line width (FWHM) are also reported in the same Figure.

In Figure S4, the average energy thresholds calculated for microdroplet laser at varying the liquid solution employed as gain medium are reported. The threshold energies have been determined from the trend of the emission peak intensity as a function of the pump energy, as reported in Figure 3 of the main text. We do not observe significative variations with the concentration of scattering materials. The differences are included in the error bars.

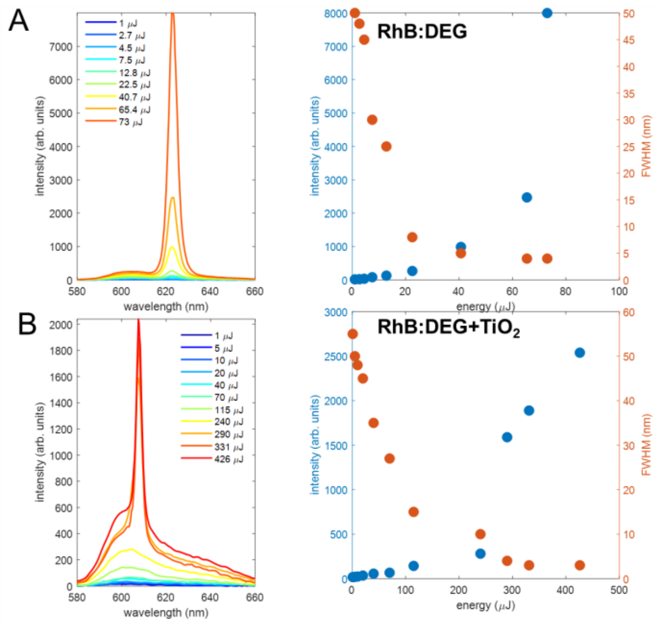

Figure S3. Emission spectra of different microlasers at varying the pump energy: RhB:DEG **A** and RhB:DEG+TiO_2 **B**. The spectra are acquired with a 600 gr/mm diffraction grating. On the right part of the figure the trends of the peak intensity (in blue) and of the FWHM (in orange) are reported.

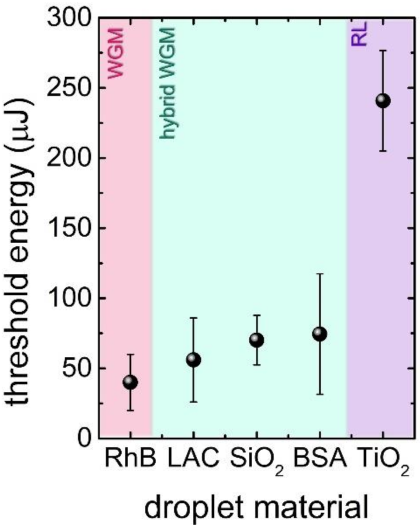

Figure S4. Average values of the lasing energy thresholds calculated for microdroplet lasers obtained with different liquid solutions. The error bars represent the corresponding standard deviations. The three different mechanisms of emission are represented in the Figure

S4. Evaluation of the limit of detection

To evaluate the limit of detection (LOD) we focused on the linear region of the trends of Figure 5 of the manuscript, corresponding to the lowest concentration analyzed, as reported in Figure S5 for both the volume fraction ϕ and for the variation in the effective refractive index Δn of the microdroplets. The LOD for each dielectric compound has been determined from the slope s obtained by the linear fitting according to the equation: LOD=3σ/s, where σ is the standard deviation of the blank[1]. We consider σ=0.07 nm, corresponding to the spectral resolution of the spectrometer. The obtained results of LODs for volume fraction ϕ, concentration c and refractive index variation Δn, expressed in refractive index units (RIU), are reported in Table S2.

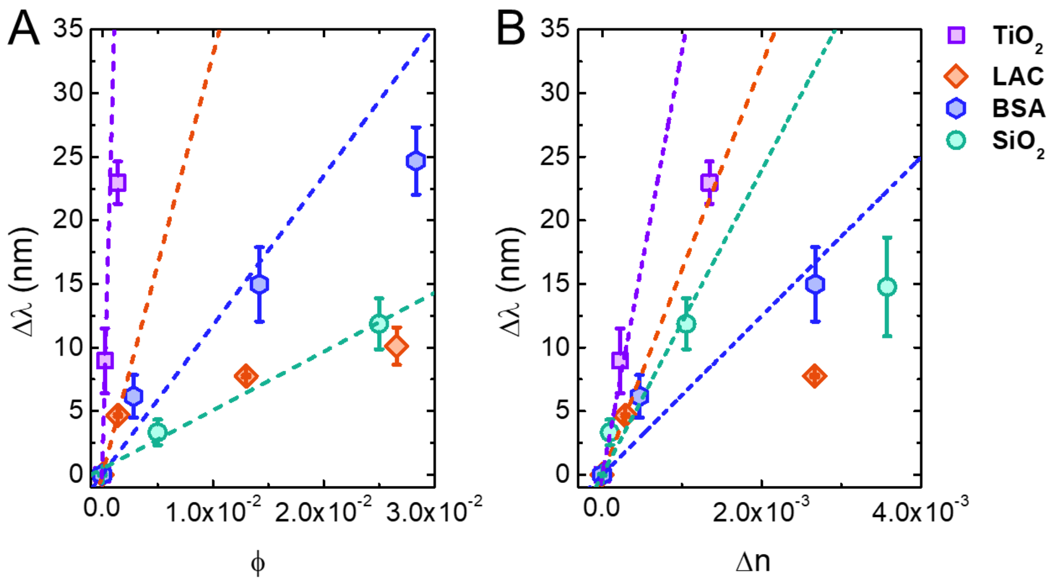

Figure S5. **A** Shift of the emission wavelength $\Delta\lambda$ of the microdroplet laser as a function of the volume fraction ϕ of the dielectric compounds dispersed in the gain medium. **B** Shift of the emission wavelength $\Delta\lambda$ of the microdroplet laser as a function of the variation in the effective refractive index Δn of microdroplets. The dashed lines in the figures represents the best linear fits for the estimate of the LOD.

The slope of the linear trends of Figure S5 B yields the sensitivity $\frac{\Delta\lambda}{\Delta n}$ with respect to the refractive index of the microdroplet laser.

Table S2. Limit of detection (LOD) calculated for volume fraction ϕ, concentration c, refractive index Δn, and sensitivity for all the dielectric compounds employed for the microdroplet lasers.

**dielectric material**	**LOD_{ϕ}**	**LOD_c [μg/mL]**	**$LOD_{\Delta n}$ [RIU]**	**sensitivity [nm/RIU]**
**TiO_2**	$6.65\cdot10^{-6}$	16	$6.31\cdot10^{-6}$	33300
**SiO_2**	$4.56\cdot10^{-4}$	1207	$1.75\cdot10^{-5}$	12000
**LAC**	$6.36\cdot10^{-5}$	112	$1.35\cdot10^{-5}$	16000
**BSA**	$1.78\cdot10^{-4}$	250	$3.36\cdot10^{-5}$	6250

References

[1] G. L. Long & J. D. Winefordner, *Analytical chemistry* **1893**, *55*, 712A-724A.
